# Supplementary material for: Modelling the effect of curves on distance running performance
Source: PeerJ. 2019 Dec 20;7:e8222. doi: 10.7717/peerj.8222 (PMC6927354; doi:10.7717/peerj.8222)
Supplement: Supplemental Information 1 [file peerj-07-8222-s001.docx]

**Appendix 1**

**What is the best strategy for running around city blocks?**

Road racers are free to choose the turn radius they prefer, but an athlete who strives to run the shortest possible distance must negotiate 0.3m radius turns. For road racing, it is important to consider the benefit of a tight radius (i.e. shortest distance) vs. the negative effect on the forces required and the consequent metabolic energy cost.

Consider a runner who is negotiating one corner, whose sides, measured along the curbs/walls, are A and B respectively (see fig. 6).


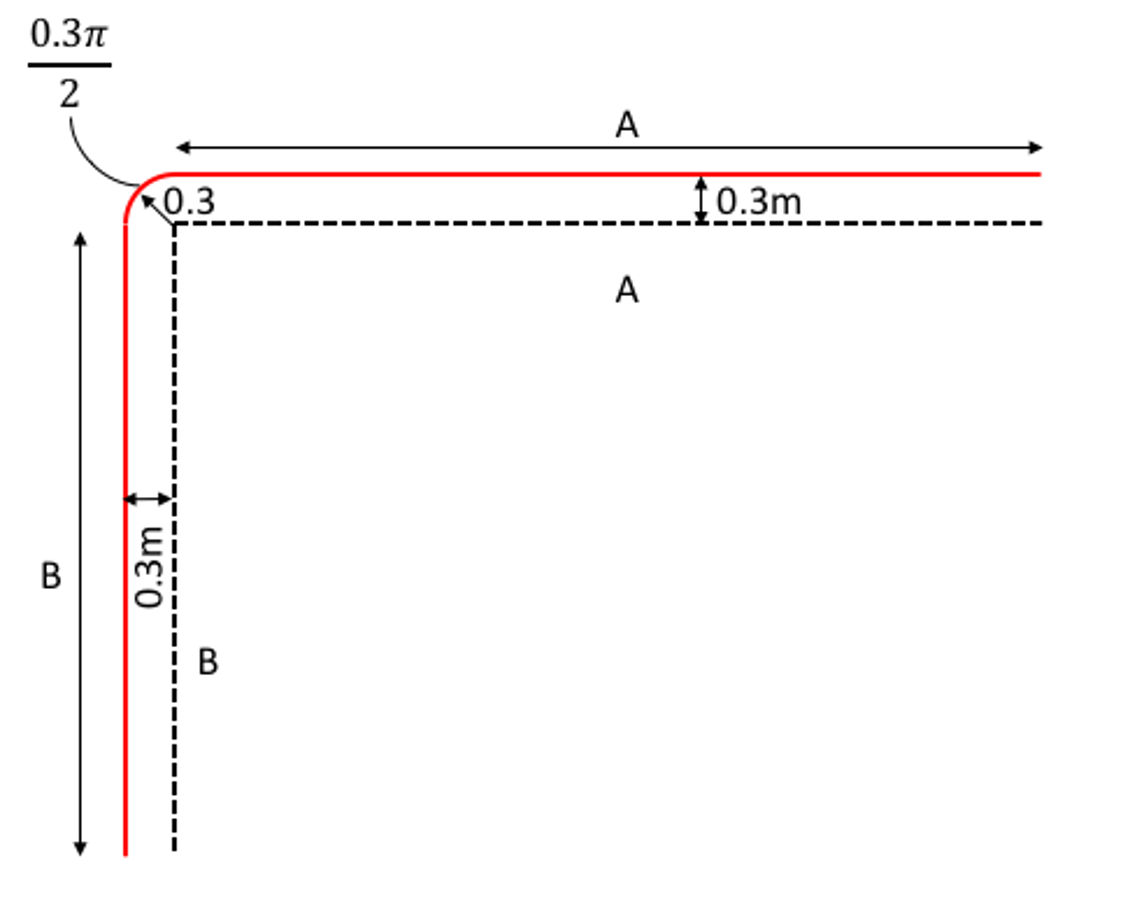


Figure 6. Dashed line: curbs/walls, red line: course measurement according to IAAF rules (IAAF 2018)

According to IAAF rules (IAAF 2018), the minimum distance from the curbs/walls is 0.3m. Therefore, the total (official) distance covered by the runner around one corner will be:

$d_{corner}=\left( A+B \right)+\frac{\pi r}{2}$ (Eq. 17)

where r = 0.3m.

A runner running around a generic block (4 corners), running exactly on the measured path would cover an official distance:

$d_{lap}=4\times d_{corner}=4\times\left( A+B \right)+0.6\pi$ (Eq. 18)

Note A and B represent the length of half of the respective sides of the block. When negotiating each of the four curved portions (each with a radius $r$ = 0.3m), a runner will likely reduce their running velocity $v_{c}$ in order to maintain a constant metabolic energy expenditure. However, the runner could limit the effect of centripetal force, and the velocity reduction around the four corners, by increasing the radius around the corner, and also their distance from the curb (see fig. 7).


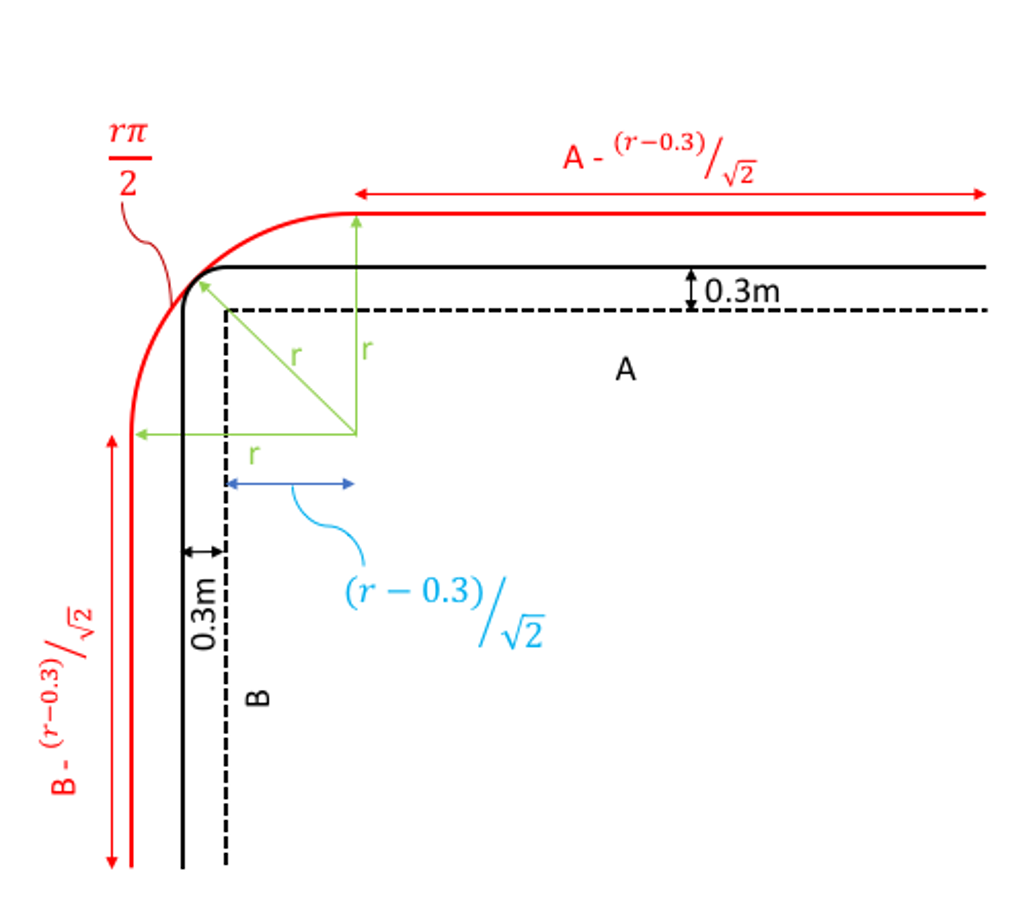


Figure 7. Dashed line indicates curb. Black line: the official path according to IAAF rules. Red line: the actual path ran by the runner.

The *actual* distance covered by the runner around one corner is:

$d_{corner}=A-\frac{\left( r-0.3 \right)}{\sqrt{2}}+B-\frac{\left( r-0.3 \right)}{\sqrt{2}}+\frac{\pi r}{2}=A+B+0.3\sqrt{2}+r\left( \frac{\pi}{2}-\sqrt{2} \right)$(Eq. 19)

Note that when $r$ =0.3 equation 19 becomes equation 17. Therefore, equation 19 represents the actual distance covered by a runner around one corner for every $r\geq0.3$.

An athlete running around a generic block (4 corners) will therefore cover an *actual* distance of:

$d_{lap, actual}=4\times\left( A+B+0.3\sqrt{2}+r\left( \frac{\pi}{2}-\sqrt{2} \right) \right)=4\times\left( A+B \right)+1.7+0.62\times r$ (Eq. 20)

While the *actual* distance will increase linearly with the radius $r$, the official distance will not.

In order to estimate the effects of different racing strategies (different radii, $r$) we considered a hypothetical marathon ran around a common US city block (e.g. Chicago (https://en.wikipedia.org/wiki/City_block)), where A= 50.3 m, B=100.6 m. One full lap would officially measure:

$d_{lap}=4\times\left( 50.3+100.6 \right)+1.9=605.5 m$ (Eq. 21)

and, in order to run a full marathon, a runner would need a total of 69.7 laps: a total distance $d_{s}$=42064 m would be run on a straight portion, while $d_{c}$=131 m would be run on a curved portion.

If a runner decided to increase the radius $r$, they would still need the same 69.7 laps, increasing their *actual* distance covered during the marathon. For example, increasing the radius by 1 m, would increase the *actual* total distance by 43.2 m. Increasing the radius would also progressively increase the portion run on the curve $d_{c}$, and decrease the portion run on the straight, $d_{s}$, up to a maximum radius $r$ = 112.8 m at which point the whole race would be on a circle ($d_{tot,actual}$=49255 m). Beyond this value, it is evident that any increase in radius would not result in any time savings due to the negligible centripetal force reduction and the overwhelming effect of total actual distance increase.

To analyze the effect of intermediate radii, we used the same approach described before. We set $v_{s}$=5.78 m/s marathon world record pace) and calculated the corresponding velocity on the curve $v_{c}$ at different radii ($0.3 m\leq r\leq112.8 m$). We then calculated the total running time taking in account the *actual* running distance to perform the 69.7 laps around the standard city block and the relative portions run on straight vs curved paths with the respective velocities $v_{s}$ and $v_{c}$. To calculate the time difference $\Delta t_{r}$ (in s) at different radii, we then subtracted the total time needed to perform the race around the block to the straight-marathon time. The best strategy to negotiate 90 degree corners is to minimize the radius r, within the aforementioned range, and thus minimize $\Delta t_{r}$. That is, when racing around a city block, runners should run as close to the curb as possible. This strategy is similar to how distance runners behave on a regular running track. Running outside of lane 1 increases the total *actual* distance and is not balanced by the negligible increase in $v_{c}$ due to the minimally reduced centripetal force, increasing the overall racing time.

**Appendix 2**

**Step-by-step algorithms**

Algorithm 1. Calculation of velocity$v_{c}$ on the curve, for a given curve radius r and gross rate of oxygen uptake

Step 1. Start with $v_{c}=v_{s}$, where $v_{s}$ is the velocity on the straight.

Step 2. Calculate ${\dot{V}O_{2}}_{s}$according to Eq. 7.

Step 3. Reduce $v_{c}$ by $\Delta v$ ( $\Delta v$ can have an arbitrary small value, in our code $\Delta v=$0.00000001

$m/s$)

Step 4. Calculate ${\dot{V}O_{2}}_{c}$ according to Eq. 8

Step 5. If ${\dot{V}O_{2}}_{c}$ > ${\dot{V}O_{2}}_{s}$ , go to Step 3,

Step 6. If ${\dot{V}O_{2}}_{c}$ ≤ ${\dot{V}O_{2}}_{s}$ , stop.

Algorithm 2. Calculation of velocity$v_{s}$ on the straight, for a given total time on the track $t_{track}$. The track has a curve radius r, $d_{s}$ and $d_{c}$ are the total distances ran on the straight and curved portions of the track, respectively, and the total racing distance is $d_{tot}=d_{s}+d_{c}$.

Step 1. Start with $v_{s}=\frac{d_{tot}}{t_{track}}$

Step 2. Calculate$v_{c}$ according to Algorithm 1.

Step 3. Increase $v_{s}$ by $\Delta v$ ( $\Delta v$ can have an arbitrary small value, in our code $\Delta v=0.0001 m/s$)

Step 4. Calculate $t_{track,new}=\frac{d_{s}}{v_{s}}+\frac{d_{c}}{v_{c}}$

Step 5. If $t_{track,new}-t_{track}>\Delta t$ go to Step 2. ($\Delta t$ can have an arbitrary small value, in our code $\Delta t=0.001 s$)

Step 6. If $t_{track,new}-t_{track}\leq\Delta t$, stop.

Note that $t_{track,new}$ takes in account the different velocities on straight vs. curved portions of the track, the goal of the algorithm is calculate $v_{s}$ and $v_{c}$ (where $v_{s}>v_{c}$) so that $t_{track,new}\cong t_{track}$.
